# Epidemiological characteristics and risk factors of obstetric infection after the Universal Two-Child Policy in North China: a 5-year retrospective study based on 268,311 cases

**DOI:** 10.1186/s12879-022-07714-7

**Published:** 2022-11-22

**Authors:** Huiqing Yuan, Cui Zhang, Ei Ni Tar Maung, Songli Fan, Zijia Shi, Fang Liao, Shuo Wang, Ying Jin, Le Chen, Li Wang

**Affiliations:** 1 Department of Obstetrics and Gynecology, Hebei General Hospital, Shijiazhuang, 050051 China; 2 Graduate School of Hebei Medical University, Shijiazhuang, 050071 China; 3 Department of Obstetrics and Gynecology, The Fifth Affiliated Hospital of Sun Yat-Sen University, No. 52 Meihua East Road, Zhuhai, 519000 Guangdong China; 4 Hebei Women and Children’s Health Center, Shijiazhuang, 050000 China; 5 Graduate School of North China University of Technology, Tangshan, 063000 China

**Keywords:** Puerperal infection, Epidemiology, Cross-sectional studys, Infection control, Only child, Family planning policy

## Abstract

**Background:**

Obstetrical infection is one of the causes of maternal death and a difficult problem for many clinicians. Changes in the demographic and obstetric background of pregnant women following the Universal Two-Child Policy may have an impact on some fertility phenomena. And with the increase in the number of deliveries, the limited medical resources become more scarce. How will China's health system quickly adapt to the growing needs and expectations for maternal health and ensure the provision of qualified and accessible medical services? In addition, what social support measures should be provided to reduce preventable obstetric complications? Given the relatively low per capita share of medical resources in China, how should China deal with the impact of the Universal Two-Child Policy? Therefore, more studies based on the change of fertility policy are needed. We try to analyze the epidemiological characteristics and risk factors of obstetric infection before and after the Universal Two-Child Policy, with a view to providing reference for the prevention and control of obstetric infection in regions after the change of fertility policy, and also hope to make corresponding contributions to the solution of the above problems through relevant studies.

**Methods:**

The subjects of the survey were 268,311 pregnant women from Hebei Province Maternal Near Miss Surveillance System (HBMNMSS) of Hebei Women and Children's Health Center from January 1, 2013 to December 31, 2017. We analyzed the region, time and population distribution characteristics of obstetric infection, compared the epidemiological factors of obstetric infection before and after the Universal Two-Child Policy, and analyzed the relevant risk factors of obstetric infection.

**Results:**

The incidence of obstetric infection increased nearly twice after the Universal Two-Child Policy. The incidence of obstetric infection was highest in Chengde (1.9%), a city with a northward geographical distribution, Baoding (1.6%), Cangzhou (1.5%) followed; The higher the hospital grade, the higher the incidence; The incidence of obstetric infections in hospitals at all levels has increased; The age of onset before the Universal Two-Child Policy was (27.82 ± 5.047) years old, and the age after the Universal Two-Child Policy was (28.97 ± 4.880) years old; The incidence of obstetric infections is higher in winter. The rate of abortion-related infection (increased from 0.61 to 1.65%) and the rate of pregnant women with high school education (increased from 0.35 to 0.74%) increased significantly. The results of multivariate Logistic regression analysis after the Universal Two-Child Policy showed that anemia (OR = 1.249, 95%CI: 1.071–1.458), chronic hypertension (OR = 1.934, 95%CI: 1.375–2.722), mild preeclampsia (OR = 2.103, 95%CI: 1.323–3.344) and severe preeclampsia (OR = 2.228, 95%CI: 1.703–2.916) were independent risk factors for obstetric infection. Gestational age ≥ 37 weeks was a protective factor.

**Conclusion:**

After the Universal Two-Child Policy, the prevention and control of obstetric infections should be strengthened, especially for abortion-related infections and elderly maternal with obstetric complications and complication in high-grade hospitals in winter. Educational background is also one of the factors that should be considered in the prevention of obstetric sensation. Prolonging gestational age is helpful to reduce the incidence of obstetric infection.

## Background

Obstetric infection is a very common problem in obstetric clinic, and it is also a difficult problem for many clinicians. According to the latest GBD (Global Burden of Disease) data for 2017, there are nearly 21 million obstetric infections worldwide [1]. Obstetric infections are also one of the major causes of maternal mortality, with puerperal sepsis caused by Streptococcus A alone responsible for more than 10% of maternal deaths worldwide, the incidence is higher in developing countries (29.99% in developing countries and 9.4% in developed countries) [2]. In a cohort study on the frequency of maternal infection in 52 countries, 6.8% of women with obstetric infections with severe complications died in hospital, with infection-related maternal deaths accounting for more than half of all deaths in hospital, and the proportion was higher in low-income countries [3]. Maternal infections can also lead to perinatal stroke, preeclampsia, stillbirth, and teratogenesis [4–6]. Obstetric infections can also aggravate existing conditions and prolong hospital stays, and studies have shown that obstetric infections continue to occur up to 6 weeks after delivery [7]. These make the limited medical resources under greater challenge.

According to the sixth census in 2010, China has a total of 139,538 million people [8], being one of the most populous countries in the world. Family Planning Policy is a basic state policy of China. China has implemented the Family Planning Policy since 1979, and this policy has prevented more than 400 million babies from being born. In 2016, with the opening of China's Universal Two-Child Policy, China announced the end of its One-Child Policy for more than 40 years [9]. Hebei Province is the middle level of economic development in China. According to China's 2010 census, Hebei Province has a population of 75,195,200 [10]. With the change of fertility policy, the rate of cesarean section, the incidence of critical diseases, sex ratio at birth and other fertility phenomena have changed [11]. The incidence of obstetric infections has also changed. This study conducted a large multi-neutral study on the epidemiological characteristics and risk factors of obstetric infection before and after the opening of Universal Two-Child Policy, which provides a basis for health decision makers and medical institutions in the prevention and control of obstetric infection.

## Methods

### The research object

This study is a multicenter cross-sectional study of cluster random sampling. The survey subjects were pregnant women from the HBMNMSS in Hebei Women and Children's Health Center on January 1, 2013 and December 31, 2017. Monitoring points follow the cluster sampling method. Hospitals with annual deliveries > 1000 cases were sampled at a ratio of 1:10, and 22 monitoring points (hospitals) in 15 counties (cities, districts) in Hebei Province were included, including provincial and municipal hospitals 7 and 15 county-level hospitals. All hospitalized cases were recorded at each monitoring site. Each monitoring hospital adopts the "Maternal Case Survey Form" designed by the China Office of Maternal and Child Health Monitoring. Professionals were selected to be responsible for filling in the form and reporting. The quality control of the reported data was carried out regularly through the provincial, municipal and county maternal and child health network to avoid missing reporting. The statistics included the cases of pregnant women who were hospitalized. All cases were final-reviewed according to the time of discharge, and cases with missing data, repeated reports, and obvious logical errors were excluded. The content of information collection includes previous pregnancy and childbirth, current childbirth, pregnancy comorbidities and complications, etc. This study was approved by ethics.

Obstetric infection is the infection that is diagnosed in the medical record according to the obstetric infection item designed in the "Maternal Case Survey Form", include abortion-related infections, uterine incision infections, urinary tract infections, upper respiratory tract infections, hrombotic phlebitis, other systemic infections/sepsis, puerperal infections. Complications and complications of pregnancy include hypertensive disease, anemia, diabetes, kidney disease, liver disease, etc. Gravidity contains this pregnancy and parity does not. Age ≥ 35 years is defined as an elderly women. The regional, temporal and population distribution characteristics of obstetric infection were analyzed to compare the changes in the incidence of obstetric infection before and after the Universal Two-Child Policy, and the related risk factors of obstetric infection were analyzed.

### Statistical analysis

A cross-sectional study was conducted on obstetric infection and related risk factors in Hebei Province. The sociodemographic and obstetric characteristics of pregnant women before and after the policy were analyzed. SPSS26.0 software was used to analyze the data. The enumeration data were expressed in terms of population and composition ratio, and the composition ratio and incidence rate were compared between groups using χ^2^ test. Multivariate logistic regression analysis was used to analyze the risk factors of obstetric infection, and P < 0.05 was considered statistically significant.

## Results

### General demographic characteristics

From January 1, 2013 to December 31, 2017, 22 monitoring hospitals in Hebei Province reported a total of 289,895 cases of pregnant women admitted to the hospital. A total of 21,584 cases with missing data, duplicate reports, and obvious logical errors were excluded, and 268,311 cases were finally included in the statistical analysis, of which 1240 cases were diagnosed as obstetric infections, with a total incidence of 0.46% (1240/268,311). The cesarean section rate at the monitoring sites was 51.1%, the vaginal delivery rate was 47%, and the abortion rate was 1.9%. There were 149,217 cases of delivery before the Universal Two-Child Policy, and 497 cases were diagnosed with obstetric infection, with an incidence rate of 0.33% (497/149,217). There were 119,094 cases of delivery after the Universal Two-Child Policy, and 743 cases were diagnosed with obstetric infection, with an incidence rate of 0.62% (743/119,094). The demographic characteristics and obstetric characteristics of pregnant women before and after the Universal Two-Child Policy are shown in Table 1.

**Table 1:** Demographic and obstetric characteristics of pregnant women before and after the Universal Two-Child Policy

Demographic and obstetric characteristics	2013–2015 (n = 149,217)	2016–2017 (n = 119,094)	χ^2^	P
Age group (years)			3927.778	< 0.01
< 20	2265 (1.52)	1310 (1.10)		
20–	34,289 (22.98)	17,999 (15.11)		
25–	69,322 (46.46)	54,919 (46.12)		
30–	31,442 (21.07)	30,761 (25.83)		
35–	9704 (6.50)	12,032 (10.10)		
≥ 40	2195 (1.47)	2073 (1.74)		
Pregnancy complications			1440.260	< 0.01
Anemia	22,412 (70.84)	47,337 (79.35)		
Diabetes	3717 (11.75)	6626 (11.11)		
Nephropathy	358 (1.13)	401 (0.67)		
Hepatopathy	446 (1.41)	693 (1.16)		
Chronic hypertension	780 (2.47)	1186 (1.99)		
Mild preeclampsia	1116 (3.53)	947 (1.59)		
Severe preeclampsia	2810 (8.88)	2464 (4.13)		
Parity			418.597	< 0.01
< 1	83,318 (55.84)	51,522 (43.26)		
≥ 1	65,899 (44.16)	67,572 (56.74)		
History of cesarean section (Times)			2905.329	< 0.01
0	122,326 (81.98)	87,390 (73.38)		
≥ 1	26,891 (18.02)	31,794 (26.62)		
Frequency of pregnancy (Times)			3557.308	< 0.01
1	68,570 (45.95)	41,158 (34.56)		
> 1	80,647 (54.05)	77,936 (65.44)		
Gestational weeks (weeks)			59.211	< 0.01
≤ 27	3662 (2.45)	3175 (2.67)		
28–	1871 (1.25)	1560 (1.31)		
33–	6626 (4.44)	5931 (4.98)		
≥ 37	137,058 (91.85)	108,428 (91.04)		
Delivery mode			31.385	< 0.01
Vaginal delivery	69,380 (46.50)	56,665 (47.58)		
Cesarean section	76,863 (51.51)	60,127 (50.49)		
Abortion	2974 (1.99)	2302 (1.93)		
Education grade			2619.692	< 0.01
Primary school and below	43,287 (29.01)	45,150 (37.91)		
Middle school	46,503 (31.16)	35,247 (29.60)		
College and above	59,427 (39.83)	38,697 (32.49)		

The data outside the bracket is the number of people, and the data inside the bracket is the constituent ratio (%); there are 7 cases missing in the obstetric infection group and 1533 cases missing in the non obstetric infection group

### Comparison of obstetric infection prevalence before and after the Universal Two-Child Policy

As shown in Table 2, the incidence of obstetric infection in all age groups after the Universal Two-Child Policy is higher than that before the Universal Two-Child Policy. The incidence of obstetric infections in the group with pregnancy complications and complications changed significantly before and after the two-child policy: chronic hypertension (increased from 1.03 to 3.46%, more than 3 times) and severe preeclampsia (from 1.42% increased to 3.33%, an increase of nearly 2.5 times). Parity ≥ 1 obstetric infection increased nearly 2 times (from 0.33 to 0.61%), and Caesarean section with 0 increased more than 3 times (from 0.30 to 1.09%). According to the gestational week group, the incidence of obstetrical infection increased more than twice in the ≤ 27 week group (from 0.76 to 1.80%). Before and after the comprehensive two-child policy, the gestational weeks with high incidence rates were both 28–32 weeks group. Grouped by delivery method, it was found that abortion-related infections (increased from 0.61 to 1.65%) had the largest increase, an increase of more than 2.7 times, and the incidence of abortion-related infection was high. The incidence of obstetric infection in winter (from 0.42 to 0.91%) was found to increase the most, more than twice as much as before, and the incidence of obstetric infection was the high in winter. Grouped by education level, it was found that the incidence of obstetric infections in high school education (increased from 0.35 to 0.74%) increased the most, more than 2 times.

**Table 2:** Comparison of prevalence of obstetric infection before and after Universal Two-Child Policy

Demographic characteristics	Obstetric infection rate 2013–2015 n1/n2 (%)	Obstetric infection rate 2016–2017 n1/n2 (%)	χ^2^	P
Age group (years)			102.256	< 0.01
< 20	0.31	0.69		
20–	0.37	0.62		
25–	0.29	0.58		
30–	0.38	0.65		
35–	0.31	0.72		
≥ 40	0.59	0.82		
Pregnancy complications			23.019	< 0.01
Anemia	0.51	0.65		
Diabetes	1.02	1.01		
Nephropathy	1.68	2.24		
Hepatopathy	1.12	1.01		
Chronic hypertension	1.03	3.46		
Mild preeclampsia	1.16	2.01		
Severe preeclampsia	1.42	3.33		
Parity			102.287	< 0.01
< 1	0.33	0.59		
≥ 1	0.33	0.61		
History of cesarean section			482.601	< 0.01
0	0.30	1.09		
≥ 1	0.49	0.79		
Frequency of pregnancy			97.872	< 0.01
1	0.30	0.36		
> 1	0.56	0.62		
Gestational weeks			96.539	< 0.01
≤ 27	0.76	1.80		
28-	1.39	2.31		
33-	0.89	1.67		
≥ 37	0.28	0.48		
Delivery mode			107.480	< 0.01
Vaginal delivery	0.18	0.35		
Cesarean section	0.46	0.80		
Abortion	0.61	1.65		
Season			167.570	< 0.01
Spring	0.30	0.61		
Summer	0.25	0.50		
Autumn	0.25	0.50		
Winter	0.42	0.91		
Hospital-level			79.773	< 0.01
Primary hospital	0.11	0.34		
Secondary hospital	0.17	0.31		
Tertiary hospital	1.02	1.54		
Education grade			105.937	< 0.01
Primary school and below	0.32	0.50		
Middle school	0.35	0.74		
College and above	0.32	0.58		

n1 is the number of obstetric infections in this group and n2 is the total number of people in this group

### Comparison of obstetric infection types before and after the Universal Two-Child Policy

In the proportion of obstetric infections of various types, there was no significant change before and after the Universal Two-Child Policy. The proportion of abortion-related infection increased from 4.72 to 8.67%. The proportion of upper respiratory tract infection was higher before and after the Universal Two-Child Policy (66.34% and 59.21%, respectively). Followed by other systemic infections/sepsis (13.78%, 19.92%, respectively). See Table 3.

**Table 3:**
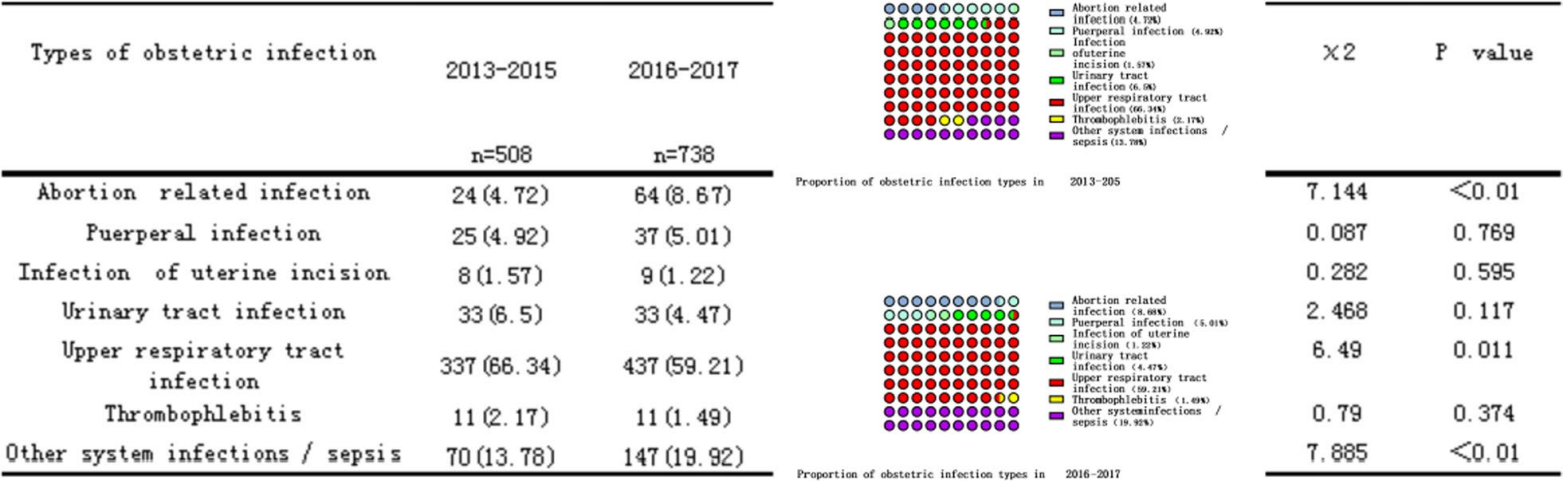
Comparison of obstetric infection types before and after the Universal Two-Child Policy

The data outside the bracket is the number of people, and the data inside the bracket is the constituent ratio (%)

This part counts the types of obstetric infections, and some pregnant women have co-infections, these pregnant women will be counted again

### Epidemiologic feature

#### Temporal distribution

**Fig. 1:**
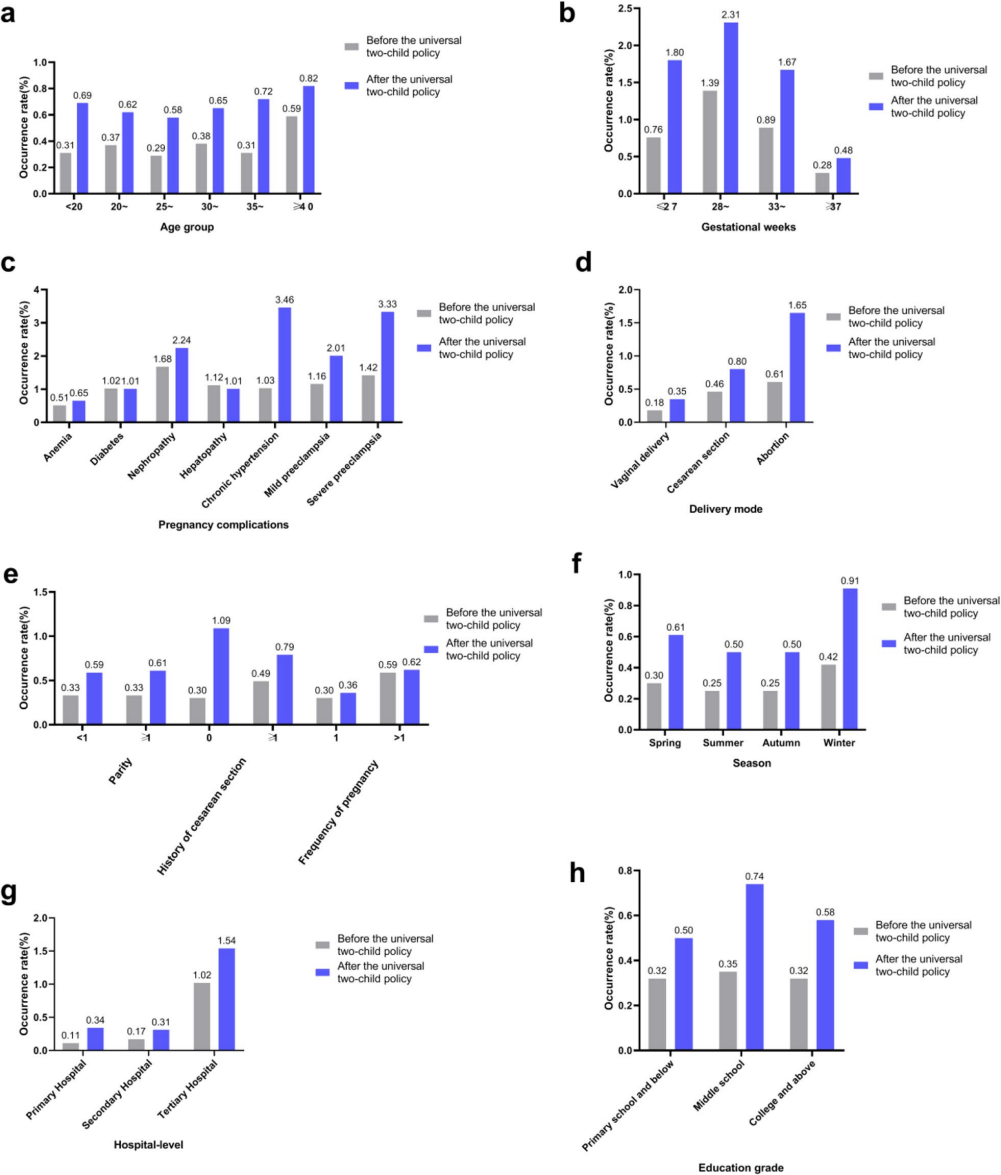
The incidence and related trends of obstetric infections in each group before and after the Universal Two-Child Policy. **a** The incidence of obstetric infections before and after the Universal Two-Child Policy by age group; **b** The incidence of obstetric infections before and after the Universal Two-Child Policy by gestational weeks group; **c** The incidence of obstetric infections before and after the Universal Two-Child Policy grouped by pregnancy complications; **d** The incidence of obstetric infections before and after the Universal Two-Child Policy grouped by mode of delivery; **e** The incidence of obstetric infections before and after the Universal Two-Child Policy grouped by parity, history of cesarean section, and frequency of pregnancy, respectively; **f** The incidence of obstetric infections before and after the Universal Two-Child Policy grouped by season; **g** The incidence of obstetric infections before and after the Universal Two-Child Policy grouped by hospital-level; **h** The incidence of obstetric infections before and after the Universal Two-Child Policy, grouped by the education grade of pregnant women

After the Universal Two-Child Policy: 187 cases (0.61%) occurred in spring (March–May), 149 cases (0.50%) in summer (June–August), 158 cases (0.50%) in autumn (September–November), and 250 cases (0.91%) in winters (December–February). There was statistical significance in the incidence between seasons (χ^2^ = 50.319, P < 0.01), and the incidence in winter was higher than that in other seasons. The overall trend before the Universal Two-Child Policy is similar to the above, but the overall incidence rate is significantly lower. As shown in Fig. 1f.

#### Regional distribution

**Fig. 2:**
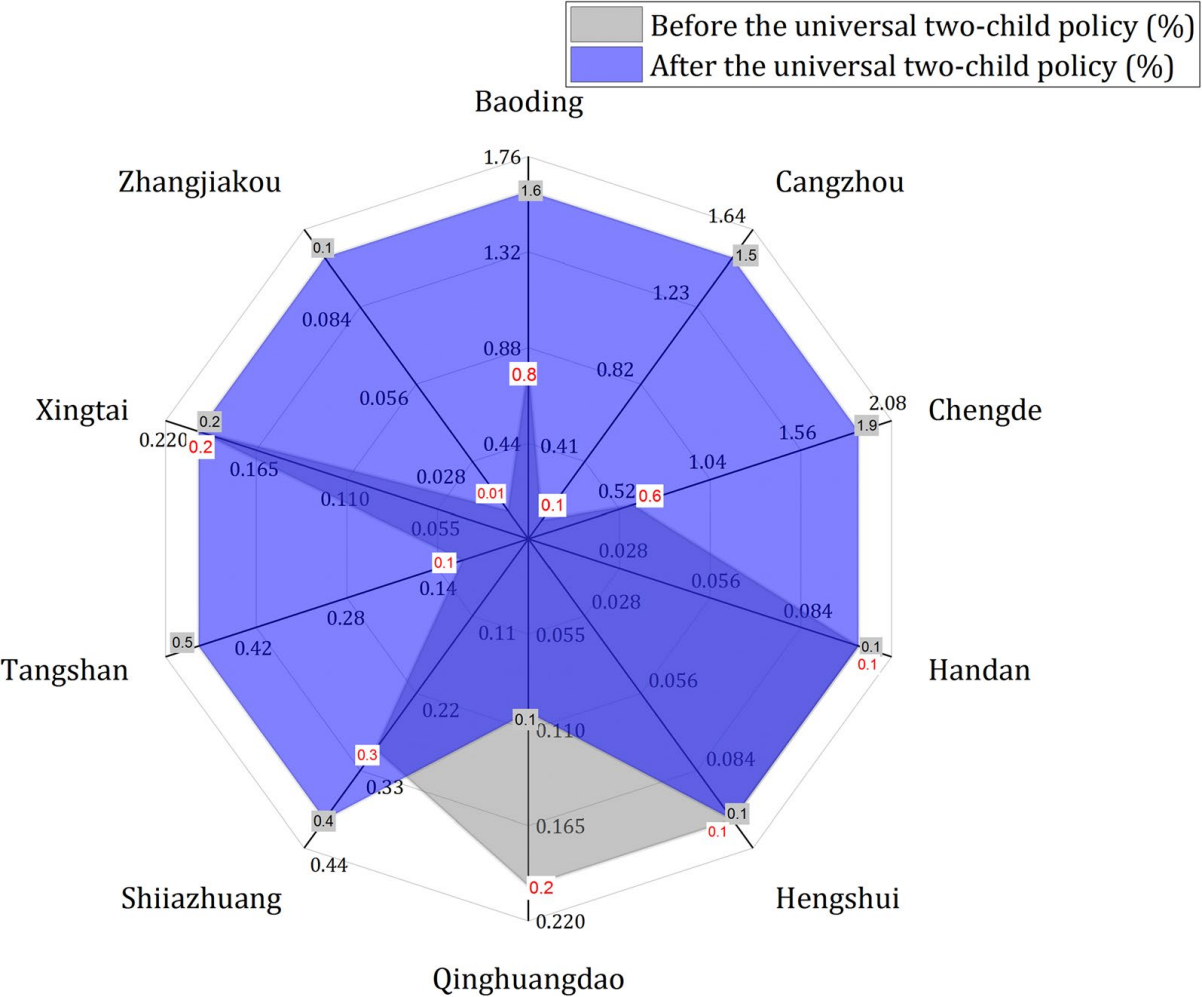
Distribution of obstetric infection areas before and after the Universal Two-Child Policy. The data filled in the white box and the gray box are the obstetric infection rate of each city before and after the Universal Two-Child Policy, respectively

The incidence of obstetric infections among pregnant women in different areas of Hebei Province is shown in Fig. 2. After the Universal Two-Child Policy, the incidence of obstetric infection in Chengde (1.9%) was the highest, followed by Baoding (1.6%) and Cangzhou (1.5%). The incidence of obstetric infection in Zhangjiakou, Qinhuangdao, Hengshui and Handan was low (all 0.1%). There was significant difference in the incidence of obstetric infection among different regions (χ^2^ = 623.060, P < 0.01). Compared with before universal two-child policy, the cities with high incidence of obstetric infection increased Cangzhou.

After the Universal Two-Child Policy, the incidence of obstetric infection at provincial and municipal level (0.77%) was significantly higher than that at county and township level (0.45%), and the difference was statistically significant (χ^2^ = 48.484, P < 0.01). The incidence of obstetric infection was 0.34% in primary hospital, 0.31% in secondary hospital and 1.54% in tertiary hospital, and there was significant difference in the incidence of obstetric infection in different levels of hospitals (χ^2^ = 541.558, P < 0.01). This was similar to the distribution of obstetric infection in hospitals at all levels before the Universal Two-Child Policy, but the obstetric infection rate in hospitals at all levels showed an upward trend after the Universal Two-Child Policy. See Fig. 1g.

### Age distribution and other corresponding distribution

Age distribution: Among the 268,311 pregnant women included in the analysis, the childbearing age before the Universal Two-Child Policy was (27.64 ± 4.518) years old, and the onset age of obstetric infection was (27.82 ± 5.047) years old. According to age group, the highest incidence was found in ≥ 40 years old group (0.59%), and the difference between age groups was statistically significant (χ^2^ = 47.837, P < 0.01). After the Universal Two-Child Policy, the childbearing age was (28.75 ± 4.547) years old, and the onset age of obstetric infection was (28.97 ± 4.880) years old. After grouping by age, the higher incidence was found in ≥ 40 years old group (0.82%), 35 ≤ age < 40 years old (0.72%), and 20 years old (0.69%). There was significant difference between the age groups (χ^2^ = 24.195, P < 0.01). As shown in Fig. 1a. Other corresponding distribution were shown in Fig. 1b, c, d, e, h.

### Multivariate logistic regression analysis of obstetric infection before and after the Universal Two-Child Policy

**Fig. 3:**
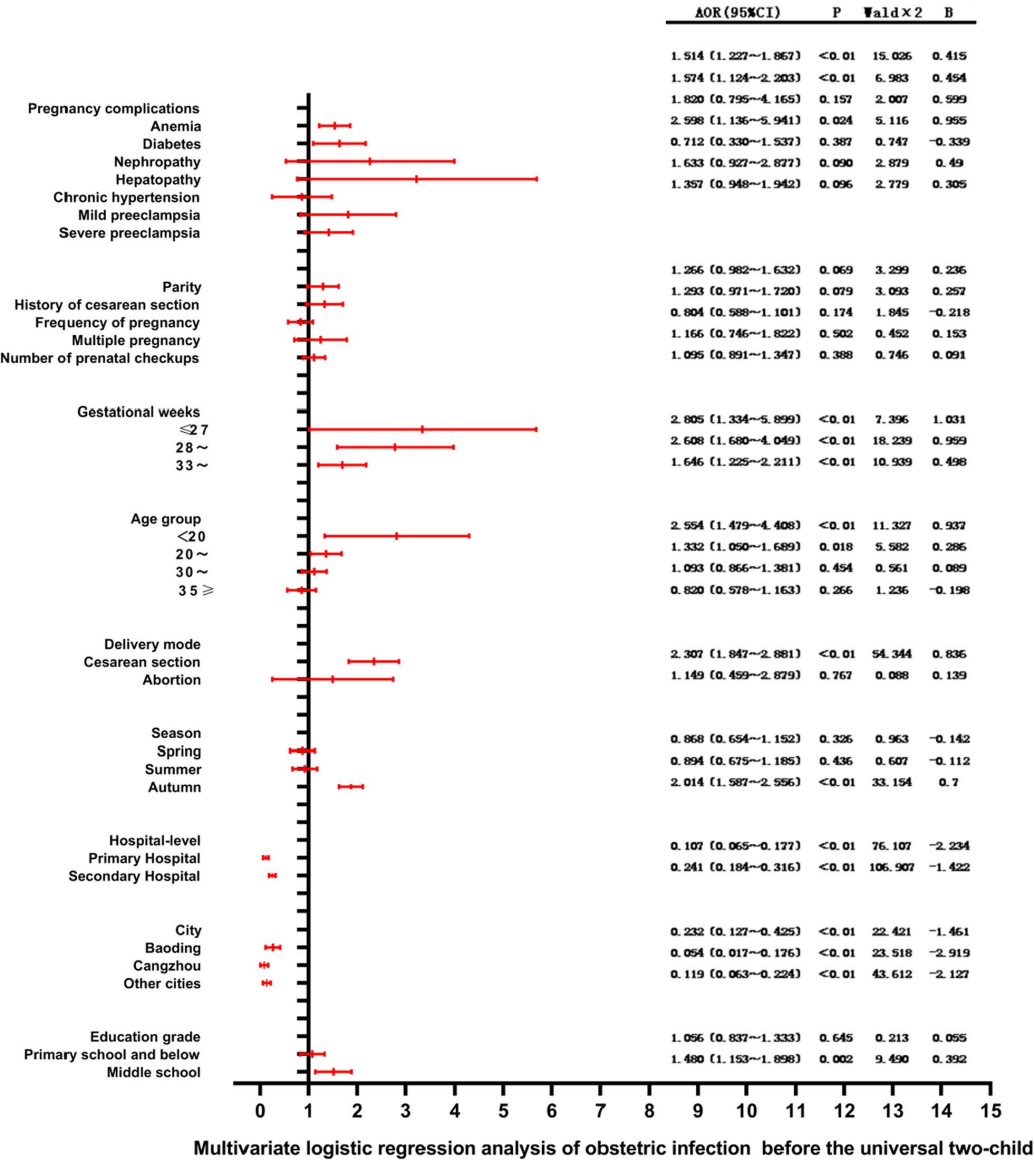

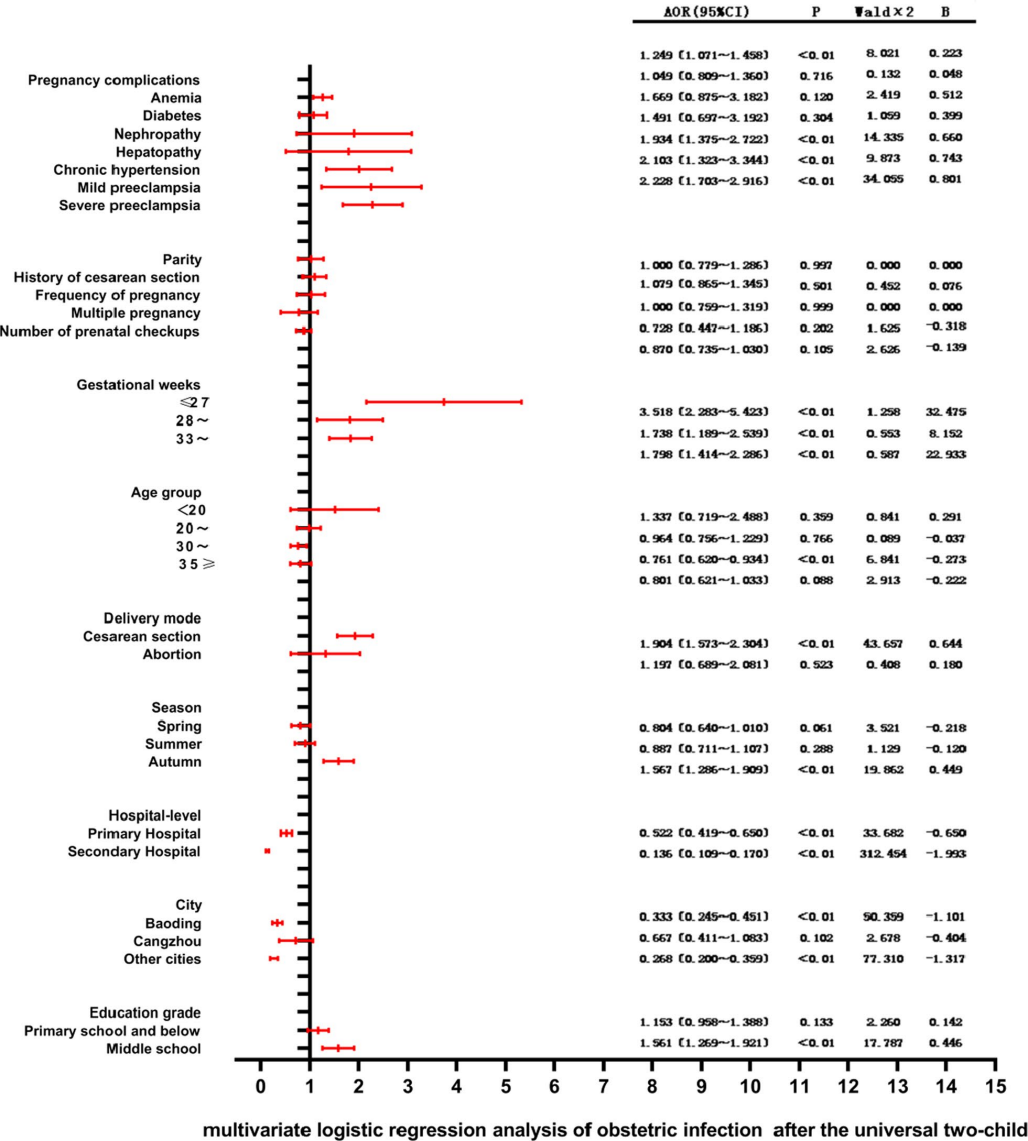
Multivariate logistic regression analysis of obstetric infection before and after Universal Two-Child Policy Hebei Province. Taking obstetric infection as the dependent variable, assignment: obstetric infection = 1, non-obstetric infection = 0. Among the independent variables, we use the independent variable assigned a value of 0 in Table 4 as the control group.

Various risk factors are associated with the incidence of obstetric infection, including anemia, diabetes [12], age [13], number of prenatal visits [14], preeclampsia [15], and history of cesarean section [16, 17]. Since season has a great relationship with influenza, and our study included upper respiratory tract infection associated with seasonal influenza, so we included season as one of the dependent variables affecting obstetric infection [18]. Caused factors for sepsis include diabetes, cardiovascular disease, eclampsia [19], location of delivery, malnutrition, first delivery, anemia, prolonged delivery time, cesarean section, and prenatal check-up times [20]. The incidence of obstetric complications varies with the level of hospital. Therefore, we used obstetric infection as the dependent variable, and factors such as anemia, diabetes, age, season, hospital level and number of prenatal visits as independent variables. The related assignments are shown in Table 4. Multivariate logistic regression analysis before the Universal Two-Child Policy showed that anemia, diabetes, age, cesarean section delivery, season, level of delivery hospital, and the city where pregnant women live affected the incidence of obstetric infections. The multivariate logistic regression analysis after the Universal Two-Child Policy showed that anemia, chronic hypertension, mild preeclampsia and severe preeclampsia were independent risk factors for obstetric infection, and age, cesarean delivery, season, hospital level and city location affected the occurrence of obstetric infection to a certain extent. Compared with the subjects ≥ 37 weeks, the subjects ≤ 27 weeks were at a higher risk from obstetric infection (HR: 2.805; 95% CI:1.334–5.889). As shown in Fig. 3.

**Table 4:** Multi-factor logistic regression analysis assignment table of factors affecting obstetric infection before and after the Universal Two-Child Policy

Project	Assignment			
Pregnancy complications				
Anemia	Yes = 1		No = 0	
Diabetes	Yes = 1		No = 0	
Nephropathy	Yes = 1		No = 0	
Hepatopathy	Yes = 1		No = 0	
Chronic hypertension	Yes = 1		No = 0	
Mild preeclampsia	Yes = 1		No = 0	
Severe preeclampsia	Yes = 1		No = 0	
Parity	> 1 = 1		1 = 0	
History of cesarean section	≥ 1 = 1		0 = 0	
Frequency of pregnancy	≥ 1 = 1		< 1 = 0	
Multiple pregnancy	Yes = 1		No = 0	
Gestational weeks	≥ 37 = 0	≤ 27 = 1	28− = 2	33− = 3
Age group	25−= 0	< 20 = 1	20− = 2	30– = 3, 35 ≥ 4
Number of prenatal checkups	≥ 8 = 0		< 8 = 1	
Delivery mode	Vaginal delivery = 0	Cesarean section = 1	Abortion = 2	
Season	winter = 0	Spring = 1	Summer = 2, Autumn = 3	
Hospital-level	Tertiary Hospital = 0	Primary Hospital = 1	Secondary Hospital = 2	
City	Chengde = 0	Baoding = 1	Cangzhou = 2, Other cities = 3	
Education grade	College and above = 0	Middle school = 1	Primary school and below = 2

## Discussion

Hebei Province, located in North China, is one of the most populous provinces. At present, there is no multi-center large-scale research on the epidemiological study of obstetric infection diseases in North China. We retrospective analyzed the data of 268,311 women hospitalized in Hebei Province in the past 5 years to explore the epidemiological characteristics and risk factors of obstetric infection before and after reproductive policy changes. This study provides information on obstetric infections in the region before and after the full two-child policy, and can be used as a useful resource for research on the prevention and control of obstetric infections after changes in the fertility policy.

### After the Universal Two-Child Policy, the incidence of obstetric infections increased, and abortion-related infections increased significantly. Educational background is also one of the influencing factors for obstetric infections after the two-child policy

The incidence of obstetric infections before and after the Universal Two-Child Policy was 0.33% and 0.62% respectively. In the era of the One-Child Policy, elective caesarean section is a common method of delivery due to various reasons [21]. At that time, various health policies in China were aimed at reducing the C-section rate, including a brief period in 2015–2016 that rewarded low C-section rates [22]. Relevant studies show that after the Universal Two-Child Policy, the average monthly caesarean delivery rate of postpartum women in China increased from 39.7 to 40.9%, an increase of 1.2 percentage points, while the average caesarean delivery rate of first-time mothers decreased from 39.6 to 36.6%, a decrease of 3.0 percentage points [23]. In a study of 7046 Chinese pregnant women, it was found that cesarean section was associated with an increased incidence of puerperium infection compared with forceps assisted delivery [24]. A systematic review and meta-analysis found that only cesarean section was positively correlated with postpartum infection [25]. Therefore, operations that do not indicate cesarean section should be avoided. In addition, due to the change of fertility policy, more and more people choose to have a second child, which may eventually encourage primipara to have vaginal delivery instead of elective caesarean delivery in order to have another child in the future [26]. Meghan A Bohren et al.'s research shows that Labour accompanys has a significant effect on maternal health during and after delivery, especially in areas with limited medical resources [27]. Therefore, increasing the delivery company may have a positive effect on reducing the incidence of obstetric infection. The abortion-related infections increased significantly, increasing to more than 2.7 times of those before the Universal Two-Child Policy. The incidence of abortion-related infection before and after the Universal Two-Child Policy was 0.61% and 1.65%, respectively, and the worldwide abortion-related infection rate was 0.1–4.7% [28]. The incidence of obstetric infection before and after the Universal Two-Child Policy was lower than the world average level of abortion-related infection rate, but the latter increased by 2.7 compared with the former. After the change of the fertility policy, the elderly pregnant are the main fertility forces. Although the Universal Two-Child Policy has greatly reduced the abortion rate of unapproved pregnancies, the early abortion rate of the older pregnant is higher [29]. Some studies suggest that infection is one of the complications associated with abortion [30]. This may be the related reason for the high incidence of abortion-related infections after the Universal Two-Child Policy. The obstetric infection rate of those with a high school education increased significantly (0.35 to 0.74%), which was more than twice the number before the Universal Two-Child Policy. In a study by Kerne-Goldberger et al., on the effect of education level on the incidence of obstetric complications, it was found that the incidence of obstetric infections, hysterectomy, uterine weakness, blood transfusion, surgical injury, arterial ligation, wound complications and intestinal obstruction were higher among pregnant women with primary and high school education. They proposed that one of the ways to reduce adverse obstetric consequences should be to increase patient consultation and improve patients' health literacy [31]. Therefore, the educational background of patients is also one of the issues that doctors should consider in preventing obstetric infection after the change of fertility policy.

### There are seasonal differences in the incidence of obstetric infections. The incidence of obstetric infections before and after the Universal Two-Child Policy is consistent with the highest in winter (0.42% and 0.91% before and after the Universal Two-Child Policy), and the incidence in other seasons is relatively stable (The Universal Two-Child Policy fluctuated between 0.25–0.30% and 0.50–0.51%, respectively)

The reasons for this analysis are as follows: in China, pregnant women give birth more often in winter [32], and winter is also the epidemic season of upper respiratory tract infection (especially influenza) [33], so the two are correlated. Moreover, studies have shown that pregnancy is believed to regulate the immune system to tolerate fetal growth, which, together with the physiological changes of pregnancy, increases the susceptibility to certain infectious diseases and also increases the mortality of influenza [34]. On the other hand, pressure can lead to decreased immunity [35, 36] and a high incidence of upper respiratory tract infection [37, 38]. In today's China, pregnant women who are pregnant with a second child are under great pressure both in their families and in the workplace. In view of the high incidence of upper respiratory tract infections in pregnant women in winter, influenza vaccination for all pregnant women during influenza season can be recommended by the Advisory Committee on Immunization Practices in the United States [39], which can protect not only pregnant women but also the fetus. Newborns are protected in the first few months of life because the anti-influenza immunoglobulin produced by the mother can be transferred to the newborn through breast milk [40, 41]. However, live attenuated vaccine is not recommended for pregnant women [42]. In addition, studies have shown that after influenza vaccination during pregnancy, the risk of several adverse perinatal outcomes (such as premature delivery, below-gestational birth rate, intrauterine fetal death, etc.) is reduced [43]. The CDC, ACIP, and ACOG also recommend that all pregnant women receive an inactivated influenza vaccine. It can be given at all stages of pregnancy, before and during flu season. The vaccine has a good safety profile and no studies have shown an association with any adverse pregnancy outcomes [44]. The reality is that the vaccination rate of pregnant women is still very low in various countries in the world [45].

### The areas with high incidence of obstetric infections before the Universal Two-Child Policy were mainly concentrated in Chengde (0.80%) and Baoding (0.60%). After the Universal Two-Child Policy, these cities are mainly concentrated in Chengde (1.90%), Baoding (1.60%) and Cangzhou (1.50%)

There is some heterogeneity among cities in Hebei Province, which means that the incidence of obstetric infection is different in some areas. Cangzhou is a petrochemical base in Hebei Province, where pollution is relatively serious, and there is a stable negative correlation between environmental pollution exposure and adverse pregnancy outcomes [46]. These pollution may have a relatively large impact on the increase of elderly maternal after the two-child policy. According to statistics, the number of births in Baoding in 2016 increased by 16,732 compared with the previous year, among which the number of one-child increased by 631, accounting for 3.78% of the total increase in births. The number of second children increased by 14,696, accounting for 87.83%; The number of multiple children increased by 1405, accounting for 8.39%. In 2017, the proportion of one child decreased, while the proportion of second, third or above children increased, and the number of second-child births exceeded that of one-child births for the first time. On the other hand, after the Universal Two-Child Policy, the second-child pregnant women are mainly elderly women born in the 70s and 80s with high risk [47]. These may be one of the reasons why Baoding has become a high-incidence city after the two-child policy [48]. Chengde is located in the northeast of Hebei Province, the climate is cold, upper respiratory tract infection (especially influenza) in the cold season, which makes Chengde become a high incidence of upper respiratory tract infection area. The upper respiratory tract infection accounted for a large proportion of obstetric infections in our statistics, which may affect the incidence of obstetric infections before and after the change of fertility policy in Chengde. In response to these changes, birth rates must be monitored locally so that the capacity of maternal and child health services can be increased as needed, thereby reducing the incidence of obstetric infections.

### The incidence of obstetric infections in hospitals of different levels was different before and after the Universal Two-Child Policy

The incidence of obstetric infection in provincial and municipal hospitals (0.77%) was significantly higher than that in county and township hospitals (0.45%), and the higher the hospital grade of childbirth, the higher the incidence of obstetric infection (Tertiary Hospital > Secondary Hospital > Primary Hospital). This is consistent with the findings of Katy et al. [49]. In general, there are significant differences in economic, cultural and educational development between urban and rural areas due to the uneven development rate of urban and rural areas in China. Cities are more developed than rural areas. As a result, the level of antenatal care and awareness of pregnancy-related care for women in urban areas may be higher than in rural areas. Compared with first-level hospitals, third-level hospitals have more advanced medical facilities and better medical technology, so they can provide patients with better standardized pregnancy management. However, this study found that the incidence of obstetric infection in provincial and municipal hospitals was higher than that in county and township hospitals. The higher the grade, the higher the incidence of obstetric infection. May be in recent years, the Chinese government to strengthen the management of severe complications of maternal, and require women with obstetric complications in tertiary hospital childbirth, so the higher the level of our country of the more critical maternal hospital, the incidence of obstetric infection relative to the higher, which may overestimate the crowd the incidence of obstetric infection in the high level hospital. A survey on the incidence of obstetric complications in the United States also found that the incidence was 5 times higher in high-grade hospitals than in low-grade hospitals [50]. There is also some evidence that hospital factors (such as teaching status, hospital geographical location, hospital level, etc.) can influence postpartum outcomes [51, 52]. Therefore, it is necessary to implement hierarchical management for high-risk groups and increase professional antenatal and intrapartum care, which are necessary conditions to ensure the successful implementation of the Universal Two-Child Policy, improve maternal and perinatal outcomes and reduce the incidence of obstetric infections.

### Our study on the age at onset of obstetric infections found a surge in advanced maternal age, an increase in the mean age at onset, and an increase in the incidence of all age groups

According to statistics, there is an increase in the number of elderly maternal in the world, and the annual increase is more than 1 million to 10 million people with many pregnancy complications and high risk [53]. After the change of China's fertility policy, there are 90 million women of childbearing age. It is estimated that 60% of women are over 35 years old and 50% are over 40 years old [54]. Statistics show that over the past three years, the number of women aged 20 to 29—the typical age range of primipara—has fallen by 3.0% from 169 million between January 2014 and June 2015 to 164 million between July 2015 and December 2016. However, in the same period, the percentage of women aged 25–34 (the typical range of postparturients) increased from 166 to 172 million, an increase of 3.6% [23]. This study showed that the proportion of elderly maternal over 35 years old increased from 7.97 to 11.84%. The age of onset before and after the Universal Two-Child Policy was (27.82 ± 5.047) years, and (28.97 ± 4.880) years. Before the Universal Two-Child Policy, the age group with high incidence of obstetric infection was ≥ 40 years old (0.59%), after the Universal Two-Child Policy, the age group with high incidence of obstetric infection was ≥ 40 years old (0.82%), 35 ≤ age < 40 years old (0.72%). After the Universal Two-Child Policy, the incidence of obstetric infections in all ages was higher than before the Universal Two-Child Policy. After the Universal Two-Child Policy, the newly increased age group with high incidence is 35 ≤ age < 40 years old, which is related to the significant increase in the proportion of pregnant women aged 35 and above after the policy [55]. Some obstetric infections are positively correlated with age [56, 57]. In the study of Sheen et al., it was found that the incidence of thrombophlebitis was higher in elderly maternal [58]. Older pregnant women are more likely to develop embryo malformations, so amniocentesis/chorionic sampling is more likely to be performed, and these two procedures are considered to be high risk factors for abortion-related infections [59]. Older women more difficult pregnancy, so make use of assisted reproductive technology pregnancy [60, 61], although an Australian study has shown that assisted reproductive technology is [62] less likely to help older pregnant women with the corresponding risk of obstetric complications than those who conceive naturally, it is undoubtedly an innovative operation that may increase the incidence of obstetric infection. With the change of the policy, the number of postpartum women nationwide exceeded the number of primiparas, that is, the increase in the birth rate was mainly driven by postpartum women who were previously restricted by the One-Child Policy [23]. This leads us to a possible conclusion that the older pregnant women and those who have delivered are more likely to be inspired by the policy to get pregnant, and these pregnant women should receive more attention from the society in the era of the "Universal Two-Child Policy".

### Multivariate logistic regression analysis of the results of this study

After the Universal Two-Child Policy, anemia, chronic hypertension, mild preeclampsia, severe preeclampsia, cesarean delivery, season, hospital level, and the city where the hospital is, to a certain extent, affect the occurrence of obstetric infections. Prolongation of gestational age is helpful to reduce the incidence of obstetric infection. Compared with the risk factors before the Universal Two-Child Policy, the risk factors associated with hypertensive diseases during pregnancy were increased. Studies have shown that the incidence of hypertensive during pregnancy in older women is increased, such as preeclampsia, which is most common in women aged 45–54 years [58]. Other studies have shown that with the increase in the number of pregnancies, hypertensive diseases during pregnancy increase [60, 63]. Ives, Christopher et al. showed that high levels of sFLT-1 (soluble FMS-like tyrosine kinase-1) and sEng (soluble internal thrombin) in patients with hypertension during pregnancy lead to abnormal immune function [64]. These findings are related to the independent risk factors for obstetric infections found in this study after the Universal Two-Child Policy was opened.

### Limitations of this study

This study was a cross-sectional retrospective investigation, and the relationship between the observed factors and obstetric infection still needs long-term longitudinal observation and related prospective studies, so as to have a more comprehensive understanding of the risk factors of obstetric infection. The survey data mainly recorded the information of pregnant and parturients during their hospitalization in obstetrics and gynecology department, and did not include outpatients, patients who visited other departments and patients who were not hospitalized. In this study, we only specialized hospitals were selected, excluding general hospitals, there may be admission rate bias. These aspects need to be further improved. But consider this study for 5 consecutive years of multicenter cross-sectional retrospective investigation, involving 22 hospitals in Hebei province, wide coverage, sample size is larger, with the crowd, the district representative, hospital level, and by repeated check and clean up the questionnaire data, authenticity and reliability of the result still can to a certain extent, reflect the policy change before and after the prevalence of obstetric infection, it has reference value.

## Conclusion

The impact of policy is a combination of many social factors (socioeconomic status, overall medical status). After the implementation of the Two-Child Policy, the incidence of obstetric infection increased significantly. There are many related factors involved in obstetric infection, and personalized measures should be taken in different regions to strengthen the popularization of knowledge about obstetric infection among women of childbearing age in prenatal, peripartum and postpartum, and to strengthen the monitoring of high-risk groups. Hospitals should also strengthen multidisciplinary management, effectively do a good job of obstetric infection prevention, diagnosis, treatment and prognosis management. Identify meaningful risk points and develop preventive measures to ensure the safety of mother and child.

## Data Availability

The datasets generated and/or analyzed during the current study are not publicly available due our research center policy, used under agreement for the current study, but are available from the corresponding author on reasonable request.

## Abbreviations

GBD: Global disease burden
sFlt-1: Fms soluble tyrosine kinase-1
sEng: Soluble intrathrombin
HBMNMSS: Hebei Province Maternal Near Miss Surveillance System
